# Role of Melanin Chemiexcitation in Melanoma Progression and Drug Resistance

**DOI:** 10.3389/fonc.2020.01305

**Published:** 2020-08-06

**Authors:** Sanjay Premi

**Affiliations:** Department of Tumor Biology, Moffitt Cancer Center & Research Institute, Tampa, FL, United States

**Keywords:** melanin chemiexcitation, reactive nitrogen species, reactive carbonyl stress, apoptosis inhibition, drug resistance

## Abstract

Melanoma is the deadliest type of skin cancer. Human melanomas often show hyperactivity of nitric oxide synthase (NOS) and NADPH oxidase (NOX), which, respectively, generate nitric oxide (NO^**·**^) and superoxide (O_2_^**·−**^). The NO^**·**^ and O_2_^−^ react instantly with each other to generate peroxynitrite (ONOO^−^) which is the driver of melanin chemiexcitation. Melanoma precursors, the melanocytes, are specialized skin cells that synthesize melanin, a potent shield against sunlight's ultraviolet (UV) radiation. However, melanin chemiexcitation paradoxically demonstrates the melanomagenic properties of melanin. In a loop, the NOS activity regulates melanin synthesis, and melanin is utilized by the chemiexcitation pathway to generate carcinogenic melanin-carbonyls in an excited triplet state. These carbonyl compounds induce UV-specific DNA damage without UV. Additionally, the carbonyl compounds are highly reactive and can make melanomagenic adducts with proteins, DNA and other biomolecules. Here we review the role of the melanin chemiexcitation pathway in melanoma initiation, progression, and drug resistance. We conclude by hypothesizing a non-classical, positive loop in melanoma where melanin chemiexcitation generates carcinogenic reactive carbonyl species (RCS) and DNA damage in normal melanocytes. In parallel, NOS and NOX regulate melanin synthesis generating raw material for chemiexcitation, and the resulting RCS and reactive nitrogen species (RNS) regulate cellular proteome and transcriptome in favor of melanoma progression, metastasis, and resistance against targeted therapies.

## Introduction

Skin is the largest human organ directly in contact with sunlight's UV rays, a potent carcinogen. Approximately 80% of the mutations in sunlight-induced melanoma are UV signature and cytosine to thymine transitions generated from cytosine-containing cyclobutane pyrimidine dimers (CPDs) (1–3). Due to its unusually wide absorption spectrum, melanin has been considered a potent shield against UV carcinogenicity. Lethal amounts of superoxide and hydrogen peroxide are generated during melanin synthesis (4, 5). Melanocytes survive this stress and remain physiologically normal in skin and in tissue culture. Exogenous factors like UV and chemical pollutants and endogenous factors like spontaneous mutations further elevate the oxidative stress (6–9), which then strongly drives melanocytic transformation into melanoma (10–12). We discovered an additional stress from endogenous activity of nitric oxide synthase (NOS) and NADPH oxidase (NOX) specifically in pigmented melanocytes and not in the isogeneic albino melanocytes (13). Literature also suggests constitutively active NOS and NOX in pigmented melanocytes and melanoma (14–16). The NO^**·**^ and ${\text{O}}_{2}^{-}$ generated, respectively, by NOS and NOX instantly combine to make peroxynitrite (ONOO^−^). ONOO^−^ induces melanin chemiexcitation, generating additional CPDs and melanomagenic mutations (13). Additionally, NOS and NOX induce post-translational modifications (PMTs) like nitration and nitrosylation that promote cellular proliferation, invasion, and drug resistance. Melanoma is an aggressive skin cancer where the cells develop resistance against targeted therapies. The role of melanin chemiexcitation, RCS, and RNS remains underrepresented in melanoma. Here we summarize that melanin-synthesis and chemiexcitation are central regulators of melanoma progression and resistance against targeted inhibitors of RAF and RAS.

## Role of Melanin Chemiexcitation in Melanomagenesis

Sunlight-induced melanoma exhibits UV signature mutations which arise from CPDs, adducts that are created spontaneously in response to UV exposure. However, we discovered that in response to UVA, the pigmented melanocytes (and not the isogenic albinos) generated CPDs for several hours in the dark, after UV exposure ended (13). Mechanistically, UV-induced reactive nitrogen species (RNS) oxidize melanin to create melanin-carbonyls in a quantum triplet state that has energy equivalent to UV photons. This energy is transferred to DNA to create CPDs in the dark (dark CPD). UV exposure also induces skin pigmentation as a screen against harmful effects of UV exposure. A probable correlation between melanin synthesis, chemiexcitation, and melanomagenesis is discussed below.

### Melanin Chemiexcitation and Melanomagenic DNA Damage

**Figure 1:**
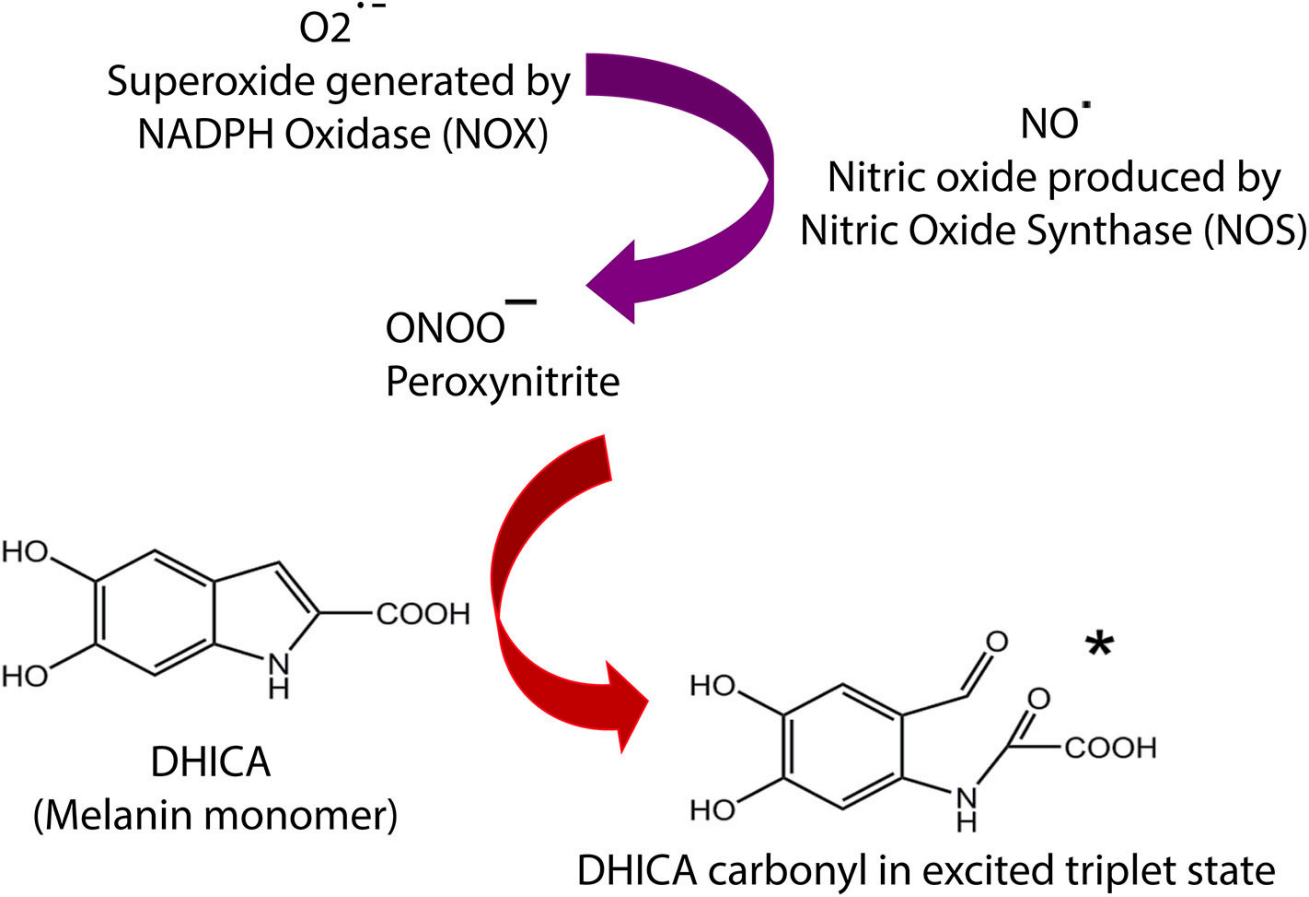
Summary of melanin chemiexcitation pathway. NOS and NOX enzymes are upregulated by UV exposure or endogenous factors, leading to generation of NO^**·**^, and O_2_^**·**^^−^, which instantly combine to produce ONOO^−^. The peroxynitrite oxidizes melanin an excited triplet state melanin-carbonyl which has energy equivalent to a UV photon. The melanin monomer 5,6-dihydroxyindole-2-carboxylic acid (DHICA) is used as an example. This carbonyl donates its energy to DNA through radiationless transfer, generating pyrimidine dimers in the dark. The remaining carbonyl molecule implements reactive carbonyl species (RNS). Complete details of the chemiexcitation pathway are explained in Premi et al. (13).

Various skin color tones (17, 18) depend upon the type of melanin and its packaging and distribution in melanosomes (19). UV-induced DNA damage and repair triggers melanin synthesis (20). Further, melanin has a wide absorption spectrum, suggesting it to be a potent screen against harmful UV rays (21) and, thus, melanoma. However, pigmented melanocytes are under constitutive oxidative stress owing to their specialized function, melanin synthesis, which generates superoxide and hydrogen peroxide (4, 5, 22–24). How melanocytes survive this oxidative stress is still debatable. Melanin itself can be pro- or antioxidant. Also, the strong intracellular redox in melanocytes is regulated by several autocrine and paracrine factors which are discussed in detail by Denat et al. (25). Adding to this, we described a contradictory, carcinogenic role of melanin in a pathway called melanin chemiexcitation (13) Figure 1. Once activated by UV exposure, the two enzymes nitric oxide synthase (NOS) and NADPH oxidase (NOX) oxidize the melanin into an excited triplet state carbonyl that donates its energy to DNA and generates carcinogenic CPDs in the complete absence of UV. This explains several contradictions in melanoma biology. For example, the most abundant wavelength, UVA in sunlight (320–400 nm), requires melanin for melanoma induction in a hepatocyte growth factor transgenic “humanized skin” mouse model (26). Similarly, spontaneous melanoma initiation was observed in *MC1R*-truncated, *BRAFV600E*-mutated mouse models (27). The *MC1R* truncation leads to higher pheomelanin/eumelanin ratio and golden coat color. Both studies suggested oxidative DNA damage as a leading factor behind melanoma development. However, we suggest a prominent contribution of protein modifications by RNS and RCS, and DNA mutations from chemiexcitation. This is supported by the endogenous NOS and NOX activity in melanocytes and >3-fold higher dark CPD in mice with golden colored (pheomelanin containing) fur (13). In addition to skin melanoma, melanin chemiexcitation might be responsible for UV signature and somatic mutations in other subtypes like acral and uveal melanoma; however, very few investigations have been done to reveal this. In 2017, Rawson et al. described UV signature mutations in ~8% cases of acral melanoma (28). Since the sites of acral melanomas are well-shielded from the sun, we predict that the UV signature in this subtype is probably due to melanin chemiexcitation. In the human eye, the cornea and iris absorb most of the wavelengths below 370 nm, which are most carcinogenic, letting through only ~2% of the longer wavelengths (370–400 nm) (29, 30). These longer wavelengths are comparatively less potent in generating CPD compared to the shorter wavelengths. Accordingly, we predict that the somatic and passenger mutations in the uveal melanoma are a result of melanin chemiexcitation. Alternatively, the UV absorption by iris and retina is not that efficient in children (31, 32), and like skin melanoma, childhood UV exposure is strongly associated with uveal melanoma at a later age. Thus, the actual role of melanin chemiexcitation in inducing melanomagenic mutation in subtypes like acral and uveal melanomas needs detailed investigations and analyses.

Despite distinct proof of the carcinogenicity of melanin, the melanin chemiexcitation pathway is still incomplete. Detailed investigation is required to identify the redox enzymes, location of melanin oxidation, route of melanin fragments to nucleus, mechanism of DNA-melanin interaction, and all the isoforms of NOS and NOX that drive melanin chemiexcitation.

Recently, we investigated if incident CPDs and the ones created by chemiexcitation can be used as genomic dosimeters for early melanoma diagnosis and better prognosis. We identified and quantified rare CPDs at a single base resolution across the genome (33). This revealed CPD hyperspots (ultra-sensitive sites) in pigmented melanocytes that had a precise alignment with the recurrent UV-signature mutations in individual gene promoters of melanomas. We also revealed that several of these are dark CPD hyperspots, created solely by the melanin chemiexcitation. Dark CPDs were confirmed by two methods. First, several sites in the pigmented human melanocytes had CPD accumulated without any UV exposure (the negative control sample) and the amount was almost equal to that of CPD induced immediately after UV exposure ended (Time 0). The same sites had none or negligible CPD in the human skin fibroblasts. Second, we collected CPD data from various time points post UV exposure. Several sites showed increased CPD amount even 7 h after UV exposure ended, compared to the CPDs immediately after UV exposure. Several genomic sites were >200-fold more sensitive for CPD generation in comparison to genome-wide average. Such hypersensitivity can alter melanocytic physiology through transcriptional blockade or epigenetic behavior in addition to the mutations.

### Pigment Type, Melanin Chemiexcitation, and Melanoma

The endogenous NOS and NOX activity is specific to pigmented melanocytes and completely absent in syngeneic albino melanocytes. We envision a physiological linkage between melanin synthesis, NOS and NOX activity, and initiation of melanoma from non-cancerous lesions like dysplastic nevi (atypical or benign moles). Nitric oxide produced either by melanocytes themselves (34) or by keratinocytes (35) upregulate the melanin synthesis pathway genes like *TYR, TRP1, DCT*, and *MITF* (34, 35). The NO^**·**^ also promotes pheomelanogenesis (pheomelanin synthesis) by modulating the *MC1R* gene expression and activity (36). A direct role of NOX in melanin-synthesis has not been demonstrated. However, the mammalian superoxide dismutase is known to oxidize L-3,4-dihydroxyphenylalanine (L-DOPA) (37), one of the initial steps in melanin synthesis. Since melanin synthesis itself produces O_2_^−^ (22), it is conceivable that melanocytes have chronic expression and activity for superoxide dismutase to eliminate O_2_^**·**^^**−**^. Thus, O_2_^**·**^^**−**^ indirectly promotes melanogenesis through DOPA oxidation by superoxide dismutase. Consequently, in the melanomagenic background, the NOS- and NOX-mediated regulation of melanin synthesis is evidently an important positive-loop that could generate raw materials for the melanin chemiexcitation pathway (13). Moreover, dysplastic nevi (atypical or benign moles) synthesize more pheomelanin compared to eumelanin (38), which is linked with dysregulated and chronic oxidative stress (39). Dysplastic nevi are known risk factors of melanoma development. Compared to eumelanogenesis (eumelanin synthesis), pheomelanogenesis is more hazardous, not only due to higher ROS production but also because it consumes cysteine, an essential component of the cellular antioxidant glutathione (40–42). We believe that the NOS and NOX activity switches eumelanogenesis to pheomelanogenesis and potentially trigger melanoma from dysplastic nevi by upregulating cellular pheomelanin content. The mechanisms behind pheomelanogenesis-mediated promotion of melanoma initiation are completely unknown. We suspect a critical role of the melanin chemiexcitation-mediated generation of CPDs, DNA mutations, microenvironment change, and melanin-carbonyls (discussed below). We believe that NOS- and NOX-mediated promotion of pheomelanogenesis is a pre-requisite for melanoma initiation from dysplastic nevi with *BRAF/NRAS* mutational background.

Our group is now investigating a NOS and NOX-regulated switch of eumelanogenesis to pheomelanogenesis and characterizes the role of this switch in initiating melanoma from non-cancerous lesions like dysplastic nevi. A direct comparison of *MC1R* polymorphism, sulfur and pheomelanin content, and melanoma markers among normal melanocytes, dysplastic nevi, and melanoma cells is envisioned to highlight the role of such a switch in the initiation of melanoma. Based on the literature and our own preliminary findings, we also believe that normal melanocytes have a physiological balance of NOS and NOX activity, chemiexcitation, and pigment synthesis. Accordingly, exo- or endogenous carcinogenesis (UV/spontaneous mutations) induces the NOS and NOX hyperactivity and altered pigment synthesis. This leads to chemiexcitation, carcinogenic dark CPDs, and altered redox in normal melanocytes either through RNS or RCS. Combined, these factors initiate melanoma from non-cancerous, *RAF/RAS*-mutated lesions.

In terms of pigment type and amount, amelanotic or hypomelanotic melanomas are outliers, however, indirectly relatable to melanin chemiexcitation. These are often present on chronically sun-exposed skin in red/blonde hair phenotypes, meaning, the ratio of pheomelanin/eumelanin is very high. This might give a lighter shade to amelanotic melanomas. Also, mortality from amelanotic melanomas is higher than the pigmented ones (43, 44). We propose that the amelanotic melanomas are rich in pheomelanin, and pheomelanin, being more potent compared to eumelanin in melanin chemiexcitation, leads to the poor prognosis of amelanotic melanomas. The misdiagnosis due to lack of color is another clinical factor responsible for poor prognosis in addition to the enhanced DNA damage and passenger mutations from melanin chemiexcitation.

### Reactive Carbonyl Species (RCS) and Melanoma

Several reactive carbonyl species have been described *in vivo* (45). We added melanin-carbonyls to this list, which are generated by NOS- and NOX-mediated chemiexcitation in pigmented melanocytes (13). After energy transfer, the melanin-carbonyls remain chemically active and induce RCS in pigmented melanocytes. Carbonyl compounds are known to deplete glutathione (46, 47). In response to a physiological UV dose, the non-melanocytic (non-pigmented) cells recover their GSH content quickly. On the contrary, pigmented melanocytes maintain a very low GSH/GSSG ratio for >4 h (Premi et al., 13). This can be attributed to either the melanin-carbonyl-mediated trapping of glutathione or the upregulation of pheomelanogenesis, which consumes cysteine. RCS cause tissue disintegration (48–50) and promote proliferative cell-signaling in several human malignancies including melanoma (50–52). Notably, the carbonyl scavengers were shown to induce pronounced apoptosis in human melanoma cell lines via the loss of mitochondrial membrane potential (53). This suggests the addiction of melanoma to the carbonyl compounds. We believe that the NOS and NOX hyperactivity, pigmentation, and resulting melanin chemiexcitation is a chronic source of melanin-carbonyls, aiding melanoma survival through unknown mechanisms. Accordingly, we classify melanin-carbonyls as potent, “non-classical” melanoma carcinogens.

Carbonyl stress can induce protein dysfunction and DNA damage. Due to their high reactivity, the well-known carbonyl sources like 4-hydroxynonenal, glyoxal, and methylglyoxal are known to interact with the free amine or sulfhydryl groups of the proteins (54–58). Accordingly, the RCS prefers arginine, cysteine, and lysine residues (59). This interaction is generally covalent and alters normal protein function (60, 61). Some studies also suggest DNA damage through glyoxal- or methylglyoxal-mediated histone glycoxidation followed by DNA strand breaks (62).

Oxidation of cellular proteins to a protein carbonyl (protein carbonylation) is frequently detected in plasma and cellular proteins in colorectal cancer patients (61, 63, 64), Hodgkin's lymphoma (65), bladder cancer (66), prostate cancer (67), and breast cancer (68). However, protein carbonylation and adduction of carbonyls to proteins are fundamentally different. The type of interaction between melanin-carbonyls and proteins/DNA is still undetermined since the chemical nature, structure, and reactivity of melanin-carbonyls is unknown. We are investigating the interaction of melanin-carbonyls with short, double-stranded DNA fragments and simple proteins like BSA in an *in vitro* chemical reaction between synthetic melanin and synthetic peroxynitrite (13). The aim is to identify DNA vs. melanin-carbonyl and protein vs. melanin-carbonyl adducts using HPLC and mass spectrometry.

### Reactive Carbonyl Species and Tumor Immunology

Melanin chemiexcitation produces an α,β-unsaturated melanin-carbonyl (13). Naturally occurring α,β-unsaturated carbonyls are immunosuppressive. Curcumin is one example that regulates JAK-STAT, AP-1, and κB in immune cells, thereby affecting the inflammatory cytokines (69, 70). Curcumin is antiproliferative for rat lymphocytes where it promotes apoptosis (71). The biology of curcumin is explained by Michael addition where its α,β-unsaturated, β-diketo moiety interacts with -SH groups on proteins through irreversible 1,4 addiction reaction (72). This also explains the antioxidant properties of curcumin where the β-diketo moiety neutralizes transition metal toxicity through chelation. Michael addition might have toxic side effects; however, it can block carcinogenesis by inducing enzymes like glutathione transferase and quinone reductase, which actively inhibit carcinogenesis.

Two other naturally occurring carbonyl compounds, chalcones and zerumbone, also induce intrinsic apoptosis in T lymphocytes by promoting the activation of caspase 3 and 9 (73–77). Chalcones are petal pigments that have α-,β-unsaturated carbonyl groups linking two aryl rings (78), and zerumbone is derived from the zingiberaceae family. A prominently anti-inflammatory chalcone that suppresses T cell proliferation is xanthohumol, which is extracted from hops (79). Several other examples of the immunosuppressive effects of α,β-unsaturated carbonyls on innate and adaptive response have been discussed in detail by Arshad et al. (80). Accordingly, the natural α,β-unsaturated carbonyls need further investigation for their therapeutic uses in autoimmune disorders. Moreover, adducts of proteins with carbonyls like malondialdehyde (MDA) and 4-hydroxynonenal(4-HNE) interfere with antibody production in pulmonary diseases (81) and several immune disorders (82–84). Adducts of formaldehyde carbonyls and proteins are known to bias the immune system toward a hypersensitive Th2 response (85). Owing to NOS–NOX hyperactivity and pigmentation, melanoma is envisioned to have a chronic melanin-carbonyl stress. No interaction of the melanin-carbonyls with proteins or other cellular molecules has been explored so far. We believe that melanin-carbonyls might suppress immune response against melanoma by modulating the tumor microenvironment. To explore this, our group is employing mass spectrometry–based strategies to first identify a “melanin-carbonyl modified” global proteome in the melanoma microenvironment and analyze the downstream effectors for their role in modulating immune response.

The participants of chemiexcitation are also known for immunomodulation. One of the prime combinatorial functions of NOS and NOX is production of ONOO^−^leading to posttranslational modifications (PTMs) like nitrosylation and nitration. Just like the chalcones and zerumbone, peroxynitrite is known to induce apoptosis in human thymocytes (86). Nitration/nitrosylation reduces the immunogenicity of peptides, antigen presentation by MHC, or TCR functions (87, 88). The RNS exposure down-regulates CD4, CD8, and chemokine receptors, thereby impairing T cell stimulation and migration (89). Nitration is known to block cytotoxic T cells from infiltrating non-melanoma tumors through sparsely known mechanisms (64, 90). In this regard, we believe that in the melanoma microenvironment, elevated NOS and NOX activity suppresses immune response through PTMs which alter TCR signaling, T cell activation, and chemotaxis. Matched detection of chemotaxis and activation markers such as CCL2, CCL5, CXCL12, CD3, CD4, CD8, CTLA-4, PD-1, and CXCR4 and nitration in melanoma tissue will establish the role of NOS and NOX in inhibiting the T cell infiltration/activation in melanoma.

## Chemiexcitation Pathway and Melanoma Drug Resistance

In addition to inducing melanin chemiexcitation and RCS in normal melanocytes, NOS and NOX enzymes are also known to be hyperactive in melanoma. Cellular pathways regulated by these enzymes are still debatable. However, scattered reports suggest their role in regulating proliferative cell signaling, apoptosis, and melanoma invasion.

### NOS and NOX Activity in Melanoma

Melanomas often show higher NOS activity (15, 16, 91, 92), which is strongly correlated with the poor survival of patients (91, 92). The well-known *BRAF* and *NRAS* mutations in melanoma (93, 94) lead to the constitutive activation of the p44/p42 MAPK pathway, which upregulates iNOS expression (95, 96). The resulting NO^**·**^ release prolongs the survival of melanoma cells by inhibiting apoptosis (16, 97, 98). The proposed mechanism for this inhibition is NOS-mediated nitration/nitrosation of Caspase 3, Hif-1α, and Phd2, conferring resistance to cisplatin (99). Additionally, NOS inhibition upregulates apoptotic proteins like Bax, Caspase-1, Caspase-3, Caspase-6, and Mdm2. The fact that this apoptosis upregulation can be prevented by inducing NOS or by exogenous chemical sources of NO^·^ (100) demonstrates the potential role of NOS in employing melanoma drug-resistance. Conversely, NOS inhibition downregulates Bcl-2, decreases intratumoral microvessel density, and increases intratumoral apoptosis *in vivo* (97, 101). Inhibiting NO^**·**^ production leads to cell cycle arrest at G_2_-M stage followed by apoptosis (100). NOX family members upregulate the redox-sensitive signaling pathways involved in tumorigenesis. NOX1 is overexpressed in melanoma cells and controls melanoma invasion by regulating matrix metaloproteinase-2 (MMP-2) transcription (102, 103). Similarly, NOX4 knockdown arrests the cell cycle at G2-M stage, thus abolishing melanoma proliferation and tumor formation (103). NOX4 also induces the phosphorylation of focal adhesion kinase, which positively modulates cell motility through cellular-Src kinase (104). In addition, Akt induction of NOX4 leads to ROS production which, along with Akt, leads to NF-κB activation, vertical growth of melanoma, and resistance to apoptosis (105, 106).

### NOS and NOX Activity and Melanoma Drug Resistance

Melanoma drug resistance is a huge clinical challenge. Several signaling pathways and secondary and tertiary mutations have been explored; however, none explain it perfectly. We believe that the NOS and NOX activity is at the center of drug resistance. There is a strong correlation between upregulated NOS and NOX activity and melanoma progression through the inhibition of apoptosis and/or promotion of cell viability. NOS- and NOX-mediated RNS induces posttranslational modifications (PTMs) like nitrosylation and nitration. We believe this regulates phosphorylation/dephosphorylation, DNA mutations, and other cellular events leading to apoptosis inhibition and drug resistance. Accordingly, inhibiting NOS–NOX will reactivate the intrinsic apoptotic pathway proteins like SMACS, Bcl-2 family, caspases, and cytochrome c. Caspases and cytochrome c are of special interest because PTMs like nitration/nitrosylation are known to inhibit their apoptotic function (107, 108). The MAPK pathway, which is constitutively activated by *BRAF* and *NRAS* mutations in melanoma, is positively associated with iNOS expression (95). Also, NF-κB is the mediator between MAPK and NOS expression (109), and NOX4 is known to induce NF-κB production (105, 106). This suggests a direct interaction and functional correlation between NOS and NOX, *BRAF*/*NRAS* mutations, melanoma progression, and drug-resistance. Irrespective of PTMs, lung cancer cells are known to survive apoptosis by triggering p53 expression in response to NO^**·**^/O_2_^−^-mediated DNA damage (110). Also, NO^**·**^-mediated upregulation of DNA-PKcs protects cells from DNA-damaging antitumor agents (111), and p53 is a major substrate for DNA-PKcs. However, this will promote clonal growth p53 mutated melanoma cells, which is not yet known. Thus, NOS- and NOX-mediated PTMs and downstream cellular events appear to be the best plausible strategy employed by melanoma cells to evade drug-mediated apoptosis.

We previously reported endogenous nuclear nitration, specifically in pigmented melanocytes, which is further elevated several-fold by UV (13). We are now investigating the physiological relevance of endogenous NOS and NOX activity in normal melanocytes to identify a baseline activity. We believe the NOS and NOX activity help melanocytes in evading apoptosis induction from the lethal oxidative stress generated during melanin synthesis. Secondly, we are identifying NOS- and NOX-induced, posttranslationally modified melanoma proteome (PMMP) and characterizing its role in cell survival, proliferation, and resistance against targeted therapies.

### Chemiexcitation Pathway and Gene Expression

The NOS- and NOX-mediated RNS, especially NO^**·**^ and ONOO^−^ are envisaged to regulate gene expression by modulating transcription factors and histone methylases/acetylases through either metal-nitrosyl complexes or direct nitration/nitrosylation (112). This is poorly defined in eukaryotic systems. In yeast, the NO^**·**^**-**mediated nitrosylation of cysteines on the transcription factor Ace1 inhibits the transcription of its target gene *CUP1* (113). NF-κB nitrosylation inhibits its DNA binding (114). NO^**·**^ also facilitates the nuclear transport of the IκBα, which displaces NF-κB from DNA and inhibits cytokine expression in endothelial cells (115). Contrary to this, NO^**·**^ stimulates IKKα activity that degrades Iκb, thereby allowing NF-κB to enter the nucleus and regulate transcription (116). NO^**·**^ ejects Zn from the zinc finger transcription factors through metal nitrosyl complexes, leading to loss of function of several transcription factors like Egr-1 and Sp1 (117, 118). In addition to affecting transcription factors, NO^**·**^ also modulates the epigenetic regulation of gene expression. Using unnaturally high doses of chemical sources of NO^**·**^ and ONOO^−^, several experimental approaches suggest an active role of NOS and NOX in regulating histone PTMs (119). Histone-modifying enzymes, particularly lysine demethylases (LSD) are amine oxidases, majority of which are Fe(II)-dependent dioxygenases that use ketoglutarate and oxygen. Nitric oxide attacks the iron core in their catalytic pocket, inhibiting demethylation and upregulating histone methylation (120). NO^**·**^-nitrosated GAPDH aids in degrading lysine methylases, thus making lysine available for acetylation. Further, NO^**·**^ activates a phosphatase which reduces global histone acetylation by activating a histone de-acetylase (HDAC) (121). On the contrary, NO^**·**^ is also known to nitrosylate cysteine residues on HDACs that blocks their binding to chromatin. These inconsistencies in the actual role of NOS and NOX in modulating gene expression has not been addressed so far. Why NO^**·**^ upregulates certain genes but suppresses others is probably explainable by either methylation or acetylation alterations. A bigger concern is chemical sources and physiologically irrelevant concentrations of NO^**·**^ being used in most of these studies. Also, most of the studies have been conducted on non-melanocytic cells. Melanomas and melanocytes have an endogenous NOS and NOX hyperactivity, which provides a unique opportunity to investigate the role of RNS and RCS in regulating gene expression in a physiological setup.

## Clinical Significance and Concluding Remarks

**Figure 2:**
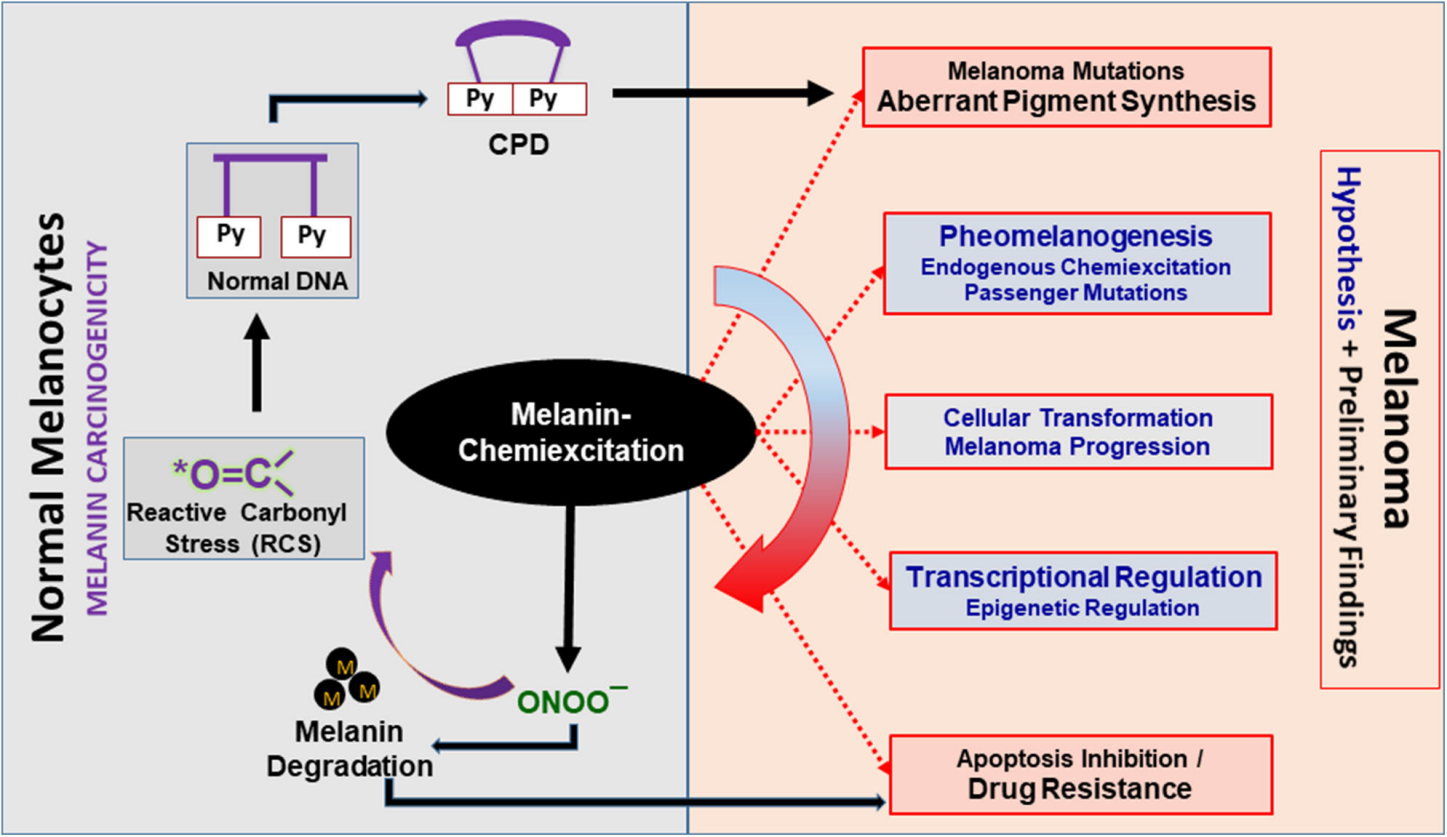
Melanin chemiexcitation as a central regulator of melanoma biology. Melanin chemiexcitation generates cyclobutane pyrimidine dimers (CPD) in the dark that induce melanomagenic mutations. In parallel, the reactive nitrogen and carbonyl species (RNS and RCS) generated during chemiexcitation regulate pigment synthesis, cellular transformations, posttranslational modifications, and gene expression. Coordinatively, this promotes melanoma progression and drug resistance through apoptosis inhibition. Black outlines have experimental proof whereas, the red outlines with gray filling and blue fonts are partially supported by experimental data. Black “M” dots represent melanin polymers, oligomers, or granules.

Based on our previous research, preliminary experiments, and the literature, it is evident that melanin chemiexcitation is a non-classical and novel pathogenesis pathway in melanoma. We believe that melanin, NOS and NOX activity, and melanin chemiexcitation are at the center of melanomagenesis and drug resistance and regulate a unique, non-classical and unidirectional positive feedback loop Figure 2. In normal melanocytes, melanin-synthesis produces lethal oxidative stress which can kill any non-melanocytic cell. However, the endogenous/physiological/baseline NOS and NOX activity protect melanocytes through PTMs and RCS. In response to an exogenous stress like UV or endogenous carcinogenic event like spontaneous mutations, NOS and NOX activity and melanin chemiexcitation are enhanced several-fold. In melanoma, the MAPK mutations like *BRAFV600E* are known to further upregulate NOS and NOX activity and expression. In parallel, the RNS upregulate melanin synthesis as a raw material for chemiexcitation that generates RCS.

In conclusion, NOS–NOX activity induce melanomagenic mutation through chemiexcitation mediated CPDs that might initiate melanoma. Secondly, the NOS–NOX activity-induced PTMs inhibit apoptogenic signaling leading to resistance to the targeted therapy. Additionally, since carbonyl scavengers induce apoptosis in melanoma, we propose that the carbonyl compounds inhibit apoptosis through unknown mechanisms. Detailed investigation of NOS and NOX activity is envisaged to either identify novel targets for new drugs or enhance the efficacy of current standard-of-care antimelanoma strategies.

## Author Contributions

SP conceived the idea, wrote the manuscript, and also made the figures.

## Conflict of Interest

The author declares that the research was conducted in the absence of any commercial or financial relationships that could be construed as a potential conflict of interest.
